# 

**Published:** 1897-06-12

**Authors:** 


					SFBPniL, UlUUUll LIlLiii.?
U U -IN K JZ Tli,
^ OADBURY'S If w, CADBURY'S
COCOA nP INI COCOA
' The standard of highest purity at present JBL <??> t NO ALKALIES USED,
attainable."?Lancet,
LASaoir Western Infibmaat
No. $5$ Vol. XXII. j fgpfper!] Saturday,
Juke 12th, 1897.
L Bee p. 176. J FRICE TWOPENCE.

				

